# Preeclampsia as a reversible risk factor for Alzheimer’s disease: A prospective MRI study on morphological changes of the cerebral cortex and impairment of cognitive functions

**DOI:** 10.1016/j.tjpad.2025.100475

**Published:** 2026-01-09

**Authors:** Yuanyuan Wang, Meng Li, Tao Chen, Yanli Li, Qingqing Wang, Xinyue Zhang, Na Wang, Linfeng Yang, Lingfei Guo, Wenying Nie

**Affiliations:** a Binzhou medical university, Guanhai Road No.346, Yantai, Shandong 264003, China; b Key Laboratory of Endocrine Glucose & Lipids Metabolism and Brain Aging, Ministry of Education; Department of Radiology, Shandong Provincial Hospital Affiliated to Shandong First Medical University, Jing-wu Road No. 324, Jinan 250021, Shandong, China; c Beijing Friendship Hospital, Affiliated to Capital Medical University, No. 95, Yong'an Road, Xicheng District, Beijing 100032, China; d Department of Psychiatry and Psychotherapy, Jena University Hospital, Germany; e Jinan Maternity and Child Care Hospital Affiliated to Shandong First Medical University, 2 Jian-guo xiao jing-san Road, Jinan, Shandong Province, China; f Heze Maternal and Child Health Hospital. No. 399, Guangzhou Road, Mudan District, Heze City 274000, Shandong Province, China

**Keywords:** Magnetic resonance imaging, Cerebral cortical thickness and surface area, Preeclampsia, Mediation analysis, Cognitive functions, Longitudinal study

## Abstract

**Purpose:**

To investigate the cross-sectional and longitudinal alterations in cortical thickness and surface area and Cognitive Impairment among patients with preeclampsia.

**Methods:**

The thickness and surface area of the cerebral cortex were systematically segmented from MRI using the automated cortical segmentation software FreeSurfer, and the corresponding values for each brain region were accurately quantified. Data collection includes the clinical characteristics, serological markers of proteins associated with cognitive function, and cognitive assessments.

**Results:**

Compared with healthy pregnancies, the cortical thicknesses of the right caudal anterior cingulate (R-CACg), right posterior cingulate (R-PoCg), right rostral anterior cingulate (R-RoACg), and right superior frontal (R-SF) in the preeclampsia group exhibited significant alterations. Notably, the change in the R-SF was specific to preeclampsia and was not associated with normal physiological pregnancies. Mediation analysis further confirmed that elevated prepregnancy BMI was directly associated with reduced Symbol Digit Modalities Test scores and indirectly contributed to cognitive decline through an increase in MAP. The cortical thickness of left pars opercularis was identified as a key component in this underlying mechanism. No statistically significant changes in cortical surface area were observed in patients with preeclampsia. Follow-up studies have indicated that cortical thickness alterations in brain regions associated with preeclampsia demonstrate signs of recovery. Among cognitive-related test indicators, only the Auditory Word Learning Test exhibited a statistically significant improvement.

**Conclusion:**

The cortical thickness alterations in the R-CACg, R-PoCg, R-RoACg, and R-SF of patients with preeclampsia may represent the structural basis for cognitive impairment. Longitudinal studies have confirmed the neuroplasticity of cortical thickness changes and the potential for recovery from preeclampsia-related memory deficits.

## Highlights

This study, for the first time, identified specific alterations in cortical thickness and cortical surface area in patients with preeclampsia. These changes closely resemble the early pathological features observed in Alzheimer's disease. The cortical thickness in brain regions, including the right posterior cingulate tail, anterior cingulate head, posterior cingulate, and superior frontal gyrus, was significantly lower in the preeclampsia group than in the PHC group. These structural changes were strongly associated with clinical factors such as elevated MAP, high prepregnancy BMI, and decreased scores on the MoCA and AVLT. These findings suggest that these cortical alterations may be involved in the pathophysiological mechanisms of cognitive impairment mediated by MAP and prepregnancy BMI. Furthermore, they may represent the structural basis underlying cognitive dysfunction in preeclampsia, providing insights into the microstructural evolution of the brain and its relationship with cognitive changes. It also may represent one of the key early alterations in brain microstructure associated with Alzheimer's disease, highlighting the importance of carefully evaluating a patient's history of preeclampsia in clinical assessment and risk stratification. Longitudinal analyses have confirmed that preeclampsia is associated with reversible changes in cortical thickness and recoverable memory decline. However, no significant recovery in overall cognitive function or associated serological markers has been reported to date.

## Graphical abstract

**Figure:**
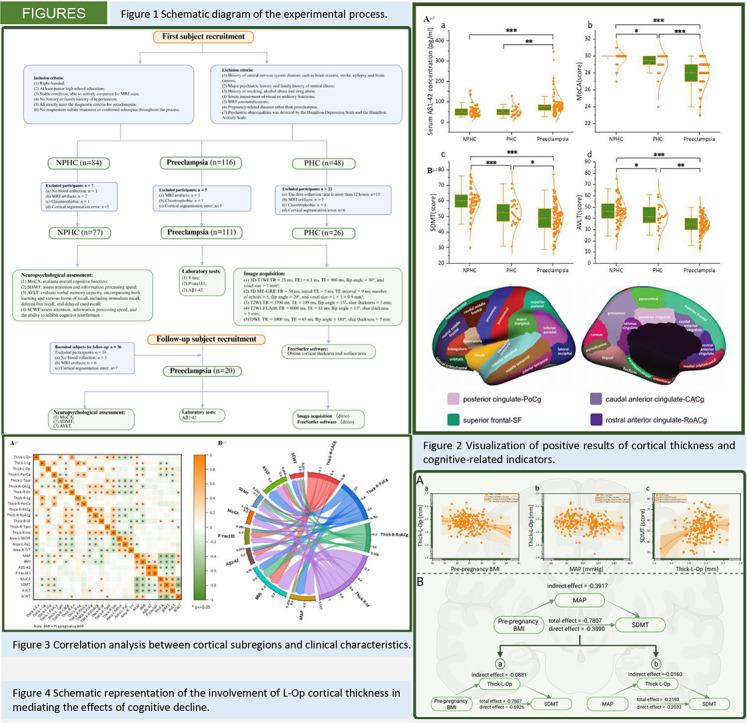

## 1 Introduction

The incidence of Alzheimer's disease among women is approximately twice that observed in men, and the majority of individuals diagnosed with dementia are female [1]. Epidemiological evidence suggests an association between gestational hypertension and an increased risk of developing Alzheimer's disease later in life. A meta-analysis indicates that women with a history of gestational hypertension face a 40 % to 92 % higher risk of Alzheimer's disease compared to those without such a history [2]. Furthermore, gestational hypertension and its severe form, preeclampsia, are linked to a 3.44-fold elevated risk of mortality due to Alzheimer's disease—a risk that surpasses those associated with diabetes, ischemic heart disease, and stroke [1]. Although gestational hypertension and Alzheimer's disease lack overlapping clinical manifestations, they exhibit notable similarities in underlying pathophysiological mechanisms [3].

Preeclampsia is a major contributor to maternal and neonatal morbidity and mortality during the perinatal period, affecting approximately 2–8 % of pregnancies globally [4]. As a placenta-derived disorder, preeclampsia involves a complex pathophysiological mechanism primarily characterized by systemic hypertension and multiorgan involvement [4]. The most severe clinical manifestations are observed in the central nervous system (CNS), where it may present with symptoms such as headache, visual disturbances, seizures, eclampsia, and stroke [4]. Epileptic episodes are typically associated with abnormal synchronous neuronal activity, which predominantly occurs in the cerebral cortex [5]. Given the potential for serious CNS complications, comprehensive research into the neurological consequences of preeclampsia and the development of effective management strategies are critically important.

This study utilized FreeSurfer software to perform a systematic and quantitative assessment of cerebral cortical thickness and surface area. Through spatial registration and anatomical segmentation of T_1_-weighted images, the software enables rapid and accurate reconstruction of the cortical surface and precise calculation of thickness and surface area across distinct brain regions. The entire analytical pipeline—including image preprocessing, cortical segmentation, surface reconstruction, and measurement—is fully automated, exhibiting high levels of consistency and reproducibility. This methodology offers robust technical support for investigating alterations in cortical thickness and surface area in patients with preeclampsia [6].

Compared with healthy pregnant women, individuals with a history of preeclampsia exhibit a significantly elevated risk of cognitive dysfunction [7], particularly in domains such as short-term and long-term working memory as well as language learning [8]. Nevertheless, current research on cognitive decline in patients with preeclampsia remains limited, especially with respect to specific impairments in memory. The limbic system comprises a network of interconnected cortical and subcortical structures, forming a functionally integrated system primarily connected by white matter tracts of varying lengths. Within this system, the Papez circuit plays a central role in integrating emotional, mnemonic, cognitive, and behavioral processes. Memory impairments are predominantly linked to structural damage in the hippocampal region of the limbic system, which is characterized by reduced cortical metabolism and decreased cortical thickness [9]. Therefore, this study hypothesizes that alterations in cortical structures within the limbic system—particularly those involving the Papez circuit—may play a crucial role in elucidating the underlying mechanisms of cognitive decline, including memory impairment, in patients with preeclampsia [1].

Accumulating evidence has revealed the presence of misfolded proteins in the serum, urine, and placenta of these patients with preeclampsia [10,11], a phenomenon that shares similarities with the pathological features observed in neurodegenerative disorders such as Alzheimer’s disease (AD). Both conditions are classified as protein misfolding disorders [12]. The core pathological hallmarks of AD include the abnormal accumulation of amyloid-β in extracellular amyloid plaques and the formation of intracellular neurofibrillary tangles resulting from the hyperphosphorylation of the tau protein [13]. β-Amyloid 1–42 (Aβ1–42) is a cleavage product of amyloid precursor protein. Owing to its neurotoxic properties, Aβ1–42 is closely linked to cognitive impairments [13]. And the abnormal phosphorylation of tau protein can impair neuronal function, thereby contributing to cognitive dysfunction—particularly in the form of phosphorylated tau protein 181 (P-tau181) [14].

This study innovatively employed cerebral cortex segmentation technology and, for the first time, revealed the association between morphological changes in the cerebral cortex and the results of laboratory examinations and cognitive neuropsychological tests. The study hypothesizes that alterations in cortical thickness may play a key role in the cognitive decline observed in patients with preeclampsia. It may also represent an imaging manifestation of early microstructural brain changes in individuals with Alzheimer's disease, potentially attributable to underlying cognitive dysfunction.

This study aims to investigate the cross-sectional and longitudinal alterations in cortical thickness and surface area among patients with preeclampsia, analyze the associated influencing factors and their correlations with cognitive decline and clinical indicators, and provide neuroimaging evidence to support early diagnosis, disease progression monitoring, and individualized treatment strategies.

## 2 Methods

### 2.1 Participants

This prospective study was reviewed and approved by the Ethics Committee of Jinan Maternity and Child Health Hospital, Affiliated with Shandong First Medical University (Approval No. 20,190,618). All participants were provided with detailed information regarding the experimental procedures and safety precautions prior to enrollment and voluntarily signed the written informed consent form.

**Fig. 1:**
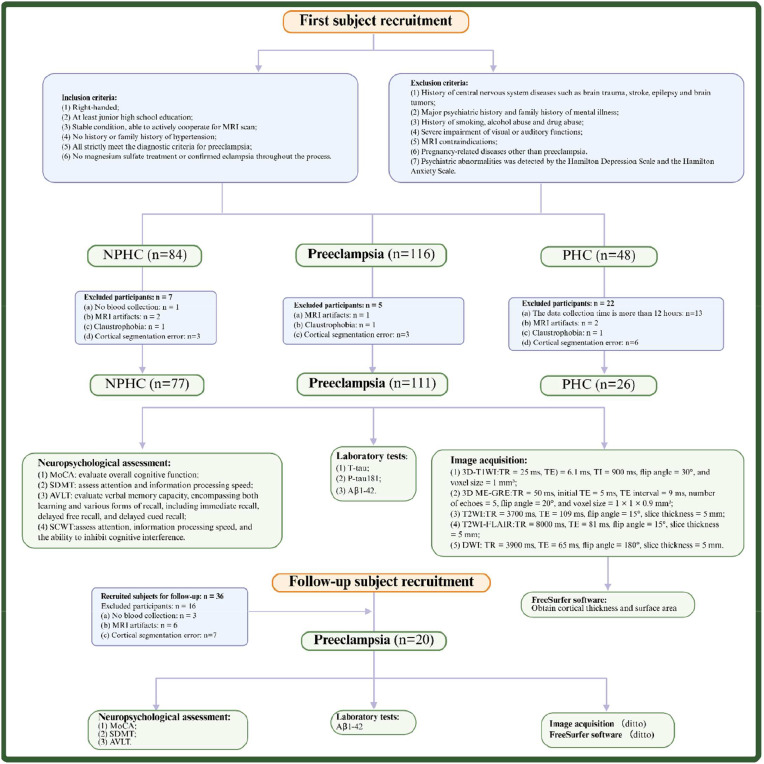
Schematic diagram of the experimental process. Following the initial recruitment of participants, they were systematically categorized based on predefined inclusion and exclusion criteria, and subsequently underwent neuropsychological assessments, laboratory testing, and MRI image acquisition and segmentation. A follow-up study was conducted three years later for participants in the preeclampsia group. Note: Serum T-tau: Serum T-tau concentration (pg/ml); Serum Aβ1–42: Serum Aβ1–42 concentration (pg/ml); Serum P-tau181: Serum P-tau181 concentration (pg/ml); MoCA: Montreal Cognitive Assessment; SDMT: Symbol Digit Modalities Test; AVLT: Auditory Word Learning Test; SCWT: Stroop Color Word Test; T_1_WI: T_1_-weighted image; T_2_WI: T_2_-weighted imaging; DWI: diffusion-weighted imaging.

A total of 111 women with preeclampsia(mean age: 31.14 ± 5.20 years; age range: 20–45 years) were enrolled in this study. These participants were recruited from the obstetric outpatient department, inpatient ward, and health examination center of the hospital between June 2021 and October 2023. The healthy control group consisted of nonpregnant healthy controls (NPHCs) (mean age: 32.21 ± 5.66 years; age range: 20–49 years; *n* = 77) and pregnant healthy controls (PHCs) (mean age: 31.04 ± 5.21 years; age range: 21–42 years; *n* = 26). We established the NPHC and PHC groups concurrently to more precisely isolate and identify changes specifically associated with pregnancy status and the pathophysiology of preeclampsia. Further details are provided in Supplementary Material 1. Three years later, follow-up examinations were conducted on the participants in the preeclampsia group between June 2024 and June 2025. The inclusion and exclusion process for follow-up participants is illustrated in Fig. 1. The reasons for incomplete data collection from certain participants are detailed in Supplementary Material 2. All participants completed data collection and underwent MRI scanning within 12 h of enrollment (the study workflow is illustrated in Fig. 1).

Inclusion criteria: (1) Right-handedness; (2) at least a junior high school level of education; (3) stable medical condition and ability to actively cooperate in completing MRI scans and neuropsychological assessments; (4) no personal or family history of hypertension; (5) all patients with preeclampsia strictly fulfilled the diagnostic criteria for preeclampsia [15]: after 20 weeks of gestation, women with previously normal blood pressure exhibited systolic blood pressure ≥140 mmHg and/or diastolic blood pressure ≥90 mmHg, or 24-hour urinary protein excretion ≥300 mg, or a urine protein/creatinine ratio ≥0.3 g/g. In cases of organ dysfunction (e.g., thrombocytopenia, renal failure, hepatic involvement, neurological symptoms, or pulmonary edema), urinary protein testing was not required; (6) no administration of magnesium sulfate treatment or confirmed diagnosis of eclampsia throughout the entire study period.

Exclusion criteria: (1) history of CNS injury, stroke, epilepsy, or brain tumor; (2) history of major psychiatric disorders or family history of such conditions; (3) history of tobacco use, alcohol consumption, or substance abuse; (4) severe visual or auditory impairment; (5) contraindications to MRI scanning; (6) patients with preeclampsia diagnosed with coexisting pregnancy-related complications other than preeclampsia, such as gestational diabetes mellitus or renal disease.

### 2.2 Clinical data collection

(1) Basic data collection: Medical records were reviewed to collect general information, medical history, and blood biochemical test results of the participants. The collected data included age, gestational weeks, height, weight, pre-pregnancy weight, systolic blood pressure, diastolic blood pressure, fasting blood glucose, glycated hemoglobin, blood lipid levels, urine microalbumin, and creatinine. Pre-pregnancy body mass index (BMI) was calculated based on height and pre-pregnancy weight, and mean arterial pressure (MAP) was derived from systolic and diastolic blood pressure measurements.
(2) Laboratory detection of cognition-related proteins: Five milliliters of anticoagulated peripheral venous blood was collected from each participant via venipuncture. Following centrifugation, serum was isolated and stored at −20 °C until analysis. Plasma concentrations of total tau protein (T-tau), phosphorylated tau protein 181 (P-tau181), and beta-amyloid protein 1–42 (Aβ1–42) were measured using enzyme-linked immunosorbent assay (ELISA, double antibody sandwich method). All assay kits were purchased from Wuhan Saipai Biotechnology Co., Ltd., and all samples were analyzed using reagents from the same batch. Each sample was tested in triplicate, and the average value was used as the final measurement.

### 2.3 Cognitive psychology tests

All participants underwent neuropsychological assessments using standardized scales. Prior to administering comprehensive cognitive function tests, their mental status was evaluated through the Hamilton Depression Scale (HAMD) and the Hamilton Anxiety Scale (HAMA) to exclude individuals with psychiatric abnormalities. All testing procedures were conducted by professionally trained medical personnel in accordance with the double-blind principle. The specific implementation details of the neuropsychological assessment are provided in Supplementary Material 3. The assessment items are as follows:

Montreal Cognitive Assessment (MoCA): comprehensive tool designed to evaluate overall cognitive function, with a maximum score of 30 points based on the number of correct responses.

(1) Symbol Digit Modalities Test (SDMT): primarily used to assess attention and information processing speed, with scores determined by the total number of correct answers.
(2) Rey Auditory Verbal Learning Test (AVLT), Huashan Version: designed to evaluate verbal memory capacity, encompassing both learning and various forms of recall, including immediate recall, delayed free recall, and delayed cued recall. The total number of correct responses is recorded as the score [16].
(3) Stroop Color and Word Test (SCWT): primarily used to assess attention, information processing speed, and the ability to inhibit cognitive interference. Participants are required to read aloud Chinese characters, colors, and the corresponding color names associated with the characters as quickly and accurately as possible, in accordance with the test instructions. The total number of correct responses is recorded as the score [17].

### 2.4 Image acquisition and preprocessing steps

The participants were scanned via a 1.5T Philips Achieva MRI scanner (Philips Healthcare) equipped with a 16-channel head phased-array coil. Anatomical structure images were obtained via a 3D-T_1_WI (T_1_-weighted image, T_1_WI) sequence for high-resolution three-dimensional brain MRI data. Conventional MRI sequences included Three-dimensional multiecho gradient-echo (3D ME-GRE), T_2_-weighted imaging (T_2_WI), T_2_WI-FLAIR, and Diffusion-Weighted Imaging (DWI), which were applied to screen for potential cerebral abnormalities. Detailed parameters of each sequence are shown in Fig. 1 and Supplementary Material 4.

Prior to each scanning session, the MRI equipment was calibrated and its performance was validated according to standardized protocols to ensure consistency in scanning parameters and the reliability of acquired image data. All participants were assessed by licensed clinical physicians, confirmed to be free from hypertensive crisis, and deemed eligible for MRI examination, with the total scanning duration approximately 17 min. Real-time monitoring was conducted throughout the imaging session to ensure participant safety and compliance.

In this study, structural MRI data were derived from T_1_-weighted and T_2_-weighted images, which were processed and corrected to ensure consistency and reliability across multiple imaging sites. T_2_-weighted images were coregistered to T_1_-weighted images by optimizing the mutual information between the two modalities to align their spatial orientation. Intensity nonuniformity correction was performed via tissue segmentation combined with sparse spatial smoothing. The images were then resampled to 1 mm isotropic voxels and rigidly aligned to a standard brain atlas. Image preprocessing and analysis were conducted via the FreeSurfer image analysis suite (version 6.0, http://surfer.nmr.mgh.harvard.edu/), implemented within fMRIPrep 20.1.1 [18,19]. FreeSurfer 6.0 is a well-validated and stable release. We selected this version to ensure direct comparability of the study results with those of previous research that has widely adopted it, and to leverage its mature and standardized processing pipelines, thereby enhancing the reproducibility of the findings and the credibility of the outcomes. Prior to image analysis, DICOM files were converted into NIFTI format via MRI conversion software. Three-dimensional T_1_-weighted images acquired via MRI underwent a series of postprocessing steps, including skull stripping to remove nonbrain tissue, intensity normalization, brain segmentation, and cortical surface reconstruction.

Following the aforementioned processing steps, researchers can generate cortical surface models of both white and gray matter. Cortical thickness was measured by FreeSurfer software as the shortest distance between corresponding vertices on the cortical surface. Topological defect correction is performed via a precise method developed by Fischl and Segonne et al., which ensures accurate correction of the cortical surface’s spherical topology [20,21]. Subsequently, images were refined on the surface and nonlinearly registered to a standardized spherical surface atlas, enabling the parcellation and labeling of cortical regions [22].

### 2.5 Statistical analysis

Statistical analysis and data visualization were conducted via IBM SPSS Statistics Version 26.0 (SPSS Inc., Chicago, IL, USA), Origin 2021, and GraphPad Prism 8. Initially, descriptive statistics were performed to summarize the demographic and clinical characteristics of the three subject groups. The Kolmogorov‒Smirnov test was applied to assess the normality of the data distribution. Continuous variables are presented as the means ± standard deviations for normally distributed data or medians ± interquartile ranges for nonnormally distributed data, whereas categorical variables are expressed as frequencies and percentages (n, %). One-way analysis of variance (ANOVA) or the chi-square test was employed to compare the clinical parameters, laboratory results, and cognitive function assessments across the three groups. In cases where significant differences were detected, Tukey’s honestly significant difference post hoc test was used for pairwise comparisons. For comparisons between two independent groups, two independent samples t tests or chi-square tests were applied, depending on the data type and distribution. Paired t tests were employed to examine longitudinal changes. Nonparametric tests were utilized when the assumption of normality was violated.

Pearson or Spearman bivariate correlation analysis was conducted to examine the relationships between cortical thickness, surface area in each brain region, and relevant clinical characteristics. The mediation analysis approach was employed to investigate the causal relationships among the variables. The total effect (TE) is defined as the sum of direct effect (DE) and indirect effect (IE). The ratio IE/TE indicates the proportion of the total effect that is mediated by cortical changes, reflecting the mediation proportion (Prop. Mediated) of M in the relationship between X and Y. Data analysis was conducted via 1000 bootstrap samples to estimate the confidence intervals.

## 3 Results

### 3.1 Clinical and cognitive characteristics

**Fig. 2:**
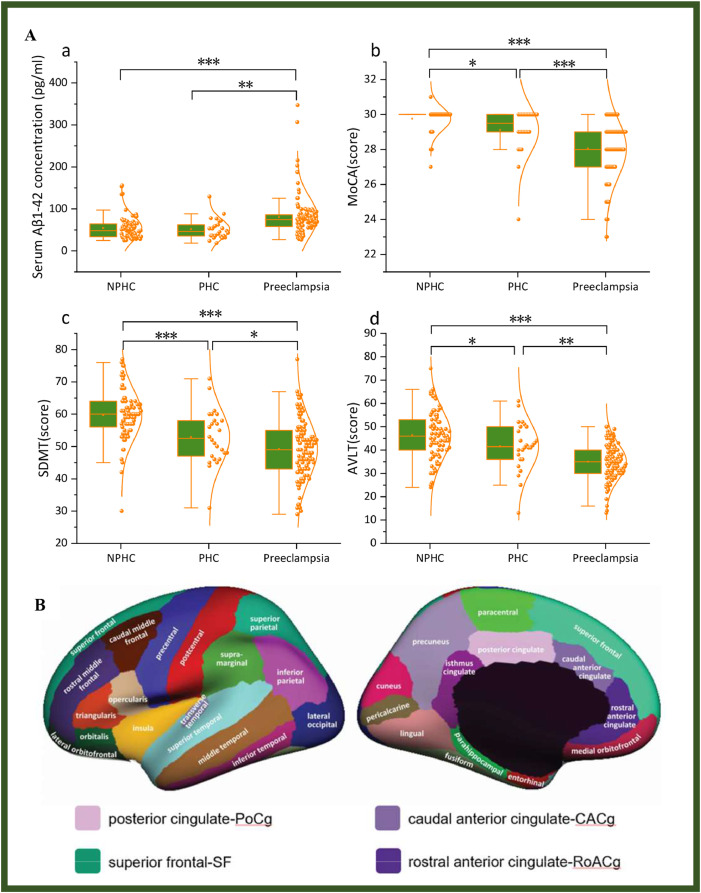
Visualization of positive results of cortical thickness and cognitive-related indicators. (A) The composite graph displaying both violin plots and box plots of cognitive-related indicators that exhibit significant inter-group differences. (B) Schematic representation of the subdivisions of the cortex of the left cerebral hemisphere, and the four brain regions highlighted in the figure represent cortical thickness alteration regions associated with preeclampsia. The original image is from https://surfer.nmr.mgh.harvard.edu/fswiki/CorticalParcellation. The illustration shows the lateral (left) and medial (right) views of the cortical surface of the left cerebral hemisphere. The unmarked area in the medial view corresponds to the non-cortical area near the anterior brain midline. Note: Serum Aβ1–42: serum β-amyloid protein 1–42 concentration; MoCA: Montreal Cognitive Assessment; SDMT: Symbol-Digit Modalities Test; AVLT: Rey Auditory Verbal Learning Test. (* P < 0.05; ** P = 0.001; *** P < 0.001).

The demographic characteristics, clinical features, laboratory findings, and neuropsychological assessment results for the three patient groups are summarized in Table S1 and Fig. 2A. Significant intergroup differences were observed in mean arterial pressure, prepregnancy BMI, serum Aβ1‒42 concentration and scores on the MoCA, SDMT, AVLT, and SCWT.

### 3.2 Changes of the cerebral cortex

The morphological changes of the 68 brain regions in the cerebral cortex are shown in Table S2. The cortical thicknesses of the right caudal anterior cingulate (R-CACg), right posterior cingulate (R-PoCg), right rostral anterior cingulate (R-RoACg), and right superior frontal (R-SF) exhibited significant intergroup differences, and the anatomical illustrations of these cortical regions are presented in Fig. 2B). The cortical thickness of the R-SF did not differ significantly among the healthy control groups, but was markedly reduced in the preeclampsia group compared to both control groups. No alterations in the surface area of the cerebral cortex associated with preeclampsia have been identified to date. It is worth noting that this finding suggests the disease's specificity may be more closely associated with a specific neuroanatomical indicator—such as cortical thickness—rather than other structural measures like surface area. This observation provides a more refined perspective on the underlying pathophysiological mechanisms.

### 3.3 Correlation analysis

**Fig. 3:**
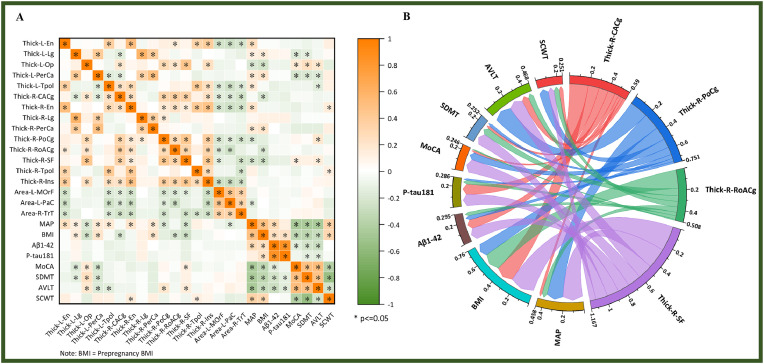
Correlation analysis between cortical subregions and clinical characteristics. (A) Heat map of correlation analysis between MAP, BMI*, cognitive function status, serological markers for cognitive function assessment (Aβ1–42 and P-tau-181) and the changes of cortical thickness and surface area. (B) Chordal plots visually demonstrate the association of the changes of cortical thickness and surface area with MAP, cognitive function status, serological markers for cognitive function assessment (Aβ1–42 and P-tau-181).

Table S3 and Fig. 3 illustrates the correlation between cortical morphological changes and clinical characteristics. The R- PoCg and R-SF were significantly correlated with MAP. Furthermore, the R-CACg, R-RoACg, and R-SF were significantly associated with prepregnancy BMI. A significant correlation was also observed between the R-SF and MoCA scores, as well as between the R-PoCg and R-SF and AVLT scores.

### 3.4 Mediation effects

**Fig. 4:**
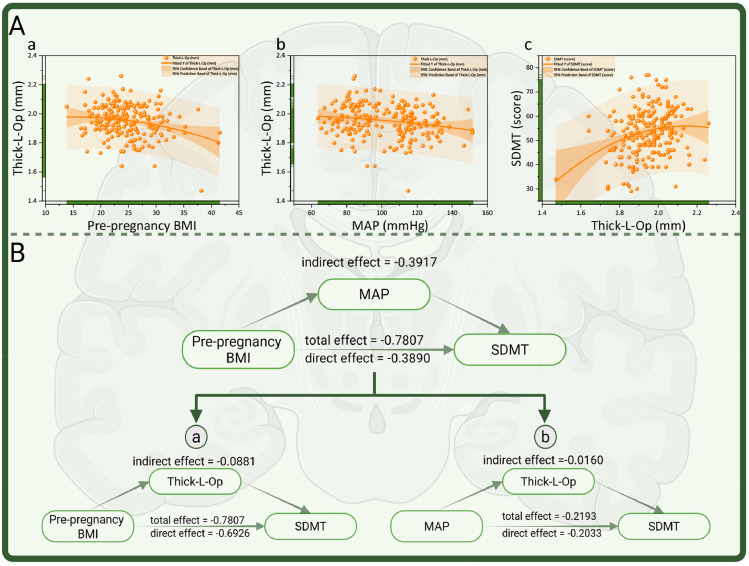
Schematic representation of the involvement of l-Op cortical thickness in mediating the effects of cognitive decline. (A) Fitted curves depicting changes in l-Op cortical thickness in women of childbearing age relative to variations in pre-pregnancy BMI (a) and MAP (b), along with the fitted curve illustrating changes in SDMT scores as a function of l-Op cortical thickness (c); (B) A schematic diagram illustrating the partial mediating role of MAP the relationship between pre-pregnancy BMI and changes in SDMT scores, mediated through the alteration of l-Op cortical thickness. a: Elevated prepregnancy BMI was directly associated with reduced SDMT scores and could also indirectly contribute to cognitive decline through a reduction in l-Op cortical thickness (DE = −0.6926, IE = −0.0881, TE = −0.7807, IE/TE ratio = 11.36 %). b: Similarly, increased MAP was directly linked to lower SDMT scores and could also exert an indirect effect by decreasing L‒Op cortical thickness (DE = −0.2033, IE = −0.0160, TE = −0.2193, IE/TE ratio = 7.30 %). Note: l-OP=left pars opercularis.

The associations between left pars opercularis (L-Op) cortical thickness and prepregnancy BMI, MAP, and SDMT score were visualized via curve fitting (refer to Fig. 4A). Mediation analysis further confirmed that elevated prepregnancy BMI was directly associated with reduced SDMT scores and indirectly contributed to cognitive decline through an increase in MAP (DE = −0.3890, IE = −0.3917, TE = −0.7807, IE/TE ratio = 50.17 %). l-Op cortical thickness was identified as a key component in this underlying mechanism (refer to Fig. 4B).

### 3.5 Longitudinal alterations in preeclampsia

**Fig. 5:**
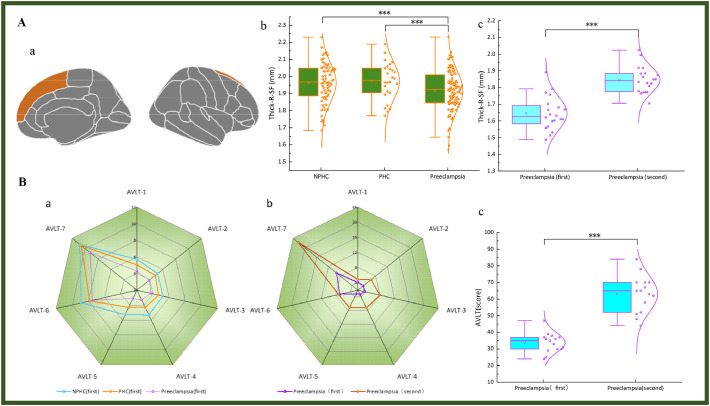
Visualization of positive results before and after follow-up of cortical thickness and cognitive-related indicators. (A) Schematic diagram illustrating brain regions exhibiting specific non-pregnancy-related changes in preeclampsia (a), along with composite violin and box plots presenting the results of post hoc ANOVA comparisons among the three groups (b), and composite violin and box plots comparing the preeclampsia group before and after a 3-year follow-up period (c). (B) Radar charts depicting the seven AVLT scores at baseline (a) and at the 3-year follow-up in the preeclampsia group, along with composite violin and box plots (c). Note: R-SF=right Sperior Frontal; AVLT=Rey Auditory Verbal Learning Test (*** P < 0.001).

The longitudinal changes in the thickness of the cerebral cortical subregions associated with preeclampsia, laboratory tests, and neuropsychological assessments in the preeclampsia group (refer to Table S4 and Fig. 5). Follow-up measurements for the R-CACg, R-PoCg, R-RoACg, and R-SF were all greater than baseline values. And follow-up laboratory test results indicated that no significant difference was observed in serum Aβ1–42 concentrations among preeclampsia participants compared to baseline values. Among the serological markers and assessments associated with cognition, only the AVLT score significantly recovered relative to the baseline values.

The results of Pearson and Spearman correlation analyses between longitudinal changes in the cortical thickness and clinical characteristics among preeclampsia participants indicated that the change in the cortical thickness of R-RoACg in preeclampsia participants, was significantly and negatively correlated with serum Aβ1–42 concentration.

## 4 Discussion

The results of this study revealed that the cortical thickness in several brain regions of patients with preeclampsia showed significant alterations compared to that in healthy pregnant women, and these changes were significantly correlated with MAP, pre-pregnancy BMI, and cognitive function-related indicators. The study hypothesizes that alterations in cortical thickness may play a key role in the cognitive decline observed in patients with preeclampsia. It may also represent an imaging manifestation of early microstructural brain changes in individuals with Alzheimer's disease, potentially attributable to underlying cognitive dysfunction.

Cortical thickness and surface area exhibit dynamic changes throughout the lifespan due to stage-specific influences. For example, both parameters undergo alterations during normal pregnancy [23]. Large-scale cohort studies in cognitively healthy community populations have demonstrated that overall and regional cortical thickness decreases at an average rate of 0.2 % per year [24]. In neurodegenerative conditions such as AD and multiple sclerosis, rapid gray matter atrophy is predominantly attributed to cortical thinning. These findings suggest that preeclampsia may accelerate age-related cortical atrophy typically observed under normal physiological conditions, and that cortical thinning may precede reductions in gray matter volume (GMV), potentially serving as a structural basis for gray matter alterations in preeclampsia.

Previous studies have substantiated, gestational hypertension disorders may be associated with structural brain alterations, including reduced cortical thickness in the temporal lobe, cingulate gyrus, and parietal lobe, as well as increased cortical surface area in the superior frontal gyrus. These brain regions are implicated in sensory and emotional integration, memory and emotional regulation, auditory and language processing, semantic memory, and emotion [25]. Furthermore, the increased cortical thickness observed in preeclampsia may reflect compensatory structural adaptations in the cortex induced by neuroinflammatory processes associated with the condition [26]. In a cohort study involving AD patients, it was found that amyloid protein deposition and hypertension may contribute to impaired cerebral perfusion through shared pathological mechanisms, particularly affecting the frontal lobe [27]. This study hypothesizes that amyloid protein deposition and hypertension-induced cerebral hypoperfusion may represent potential mechanisms underlying cortical thinning in preeclampsia. These findings suggest that interventions targeting amyloid deposition and cerebral perfusion may help mitigate cerebral injury in preeclampsia patients.

Neuroimaging studies have demonstrated that obesity can induce widespread structural alterations in the cerebral cortex. In healthy individuals with elevated BMI, increased BMI is associated with cortical thinning in the parietal, temporal, occipital, and frontal lobes. This study confirmed a significant negative correlation between pre-pregnancy BMI and cortical thickness in specific brain regions. Furthermore, resting-state functional MRI (rs-fMRI) examinations have revealed that in healthy individuals, higher BMI is linked to reduced cerebral blood flow in the prefrontal cortex, as well as decreased resting-state functional connectivity between the caudate nucleus and the prefrontal cortex. These findings suggest that altered prefrontal connectivity may represent a potential neural mechanism underlying BMI-related cognitive regulation [28,29]. Therefore, effective management of BMI in patients with preeclampsia, along with future investigations into functional connectivity and other neuroimaging features using fMRI, may hold significant value for the precise diagnosis and targeted treatment of CNS complications associated with preeclampsia.

Patients with preeclampsia exhibit abnormal dynamic cerebral blood flow, characterized by an increased resistance index, elevated perfusion pressure, and a general decline in dynamic cerebral blood flow autoregulation index [30]. Hypertension-induced reductions in global and regional cerebral perfusion are associated with decreased cortical thickness in the frontal, temporal, and parietal lobes, and are also linked to an increased risk of hypertension-related cognitive dysfunction [31]. Previous mediation analyses have indicated that elevated BMI affects brain tissue through low-grade inflammation, with obesity-related neuroanatomical changes manifesting as cognitive impairments [32] This study represents the first confirmation that pre-pregnancy BMI in women of childbearing age may influence cognitive decline through alterations in cortical structure and MAP.

The mediation analysis conducted in this study indicates that cortical thickness in the l-Op region plays a significant mediating role in the relationship between preeclampsia and cognitive dysfunction. Beyond the observed statistical association, a potential mechanistic pathway may underlie these findings, involving the following sequence of biological and neurophysiological processes: (1) Initiating factor: Placental-derived factors associated with preeclampsia, including elevated levels of inflammatory cytokines and oxidative stress byproducts, are released into the maternal systemic circulation [33,34]; (2) Central effects: These circulating factors may induce localized neuroinflammatory responses and microcirculatory disturbances in specific brain regions, such as the l-Op region, through disruption of blood-brain barrier integrity or via direct effects on cerebral vasculature and neuronal cells. Accumulating evidence indicates that a history of preeclampsia is associated with increased cerebral white matter lesions and elevated levels of neuropathological markers, including beta-amyloid protein, which are implicated in Alzheimer's disease pathogenesis [[35], [36], [37]] (3) Structural changes: Persistent neuroinflammation and disruption of the neural microenvironment may lead to reduced synaptic density, neuronal atrophy, or impaired glial support function in this brain region [[38], [39], [40]] At the macroscopic level, these cellular and structural alterations are reflected in cortical thinning. (4) Functional outcomes: Given that the l-Op region is involved in higher-order integrative functions, impairments in its structural integrity can directly compromise neural network efficiency and information processing capacity. These neurobiological changes ultimately manifest as reduced performance on cognitive assessments, particularly in domains such as memory and executive function [41,42]. Therefore, cortical thickness in the l-Op region may serve not only as a structural biomarker but also as a neuroimaging manifestation and cumulative reflection of systemic neuropathological sequelae associated with preeclampsia. It mediates the long-term impact of pregnancy-related pathological processes on cognitive decline, offering novel insights into the mechanisms underlying cognitive impairment in individuals with a history of preeclampsia and representing a potential target for early diagnosis and therapeutic intervention.

This study identified the R-SF as a specific brain region affected in preeclampsia, independent of normal pregnancy-related structural brain changes. Cortical thickness in this region was found to be positively correlated with the MoCA score. The right superior frontal gyrus constitutes a critical component of the frontal lobe and is primarily involved in the regulation of high-level cognitive functions, including executive function, attention control, spatial cognition, working memory, self-awareness, social cognition, and emotional regulation. In the preeclampsia group of this study, 11 participants (9.91 %) were diagnosed with mild cognitive impairment. Mild cognitive impairment represents a transitional phase between normal aging and dementia, characterized by neurobiological features such as hypoperfusion and hypometabolism in the temporoparietal cortex, medial temporal lobe atrophy, elevated levels of tau and phosphorylated tau proteins in cerebrospinal fluid, reduced concentrations of Aβ42 protein, and cerebral Aβ1–42 protein deposition [43], findings that closely align with those of the present study. Therefore, the specific alterations in cortical thickness of the R-SF may serve as a key therapeutic target and potential imaging biomarker for the clinical diagnosis and intervention in terms of cognitive function impairment for preeclampsia patients.

The cortical thickness of the R-PoCg and R-SF in the preeclampsia group showed a positive correlation with AVLT scores. The posterior cingulate cortex, as a key component of the limbic system, is involved in a range of high-level functions, including self-awareness and self-reflection, episodic memory and emotional integration, spatial orientation and attention, emotional regulation and response, and serves as a core region of the default mode network. It maintains strong anatomical connections with the parahippocampal gyrus and the entorhinal cortex, thereby linking to the hippocampal memory system via the dorsal pathway [44]. Impairment in amnestic mild cognitive impairment most commonly manifests as deficits in episodic memory retention and retrieval, with disruption of the posterior cingulate cortex–parahippocampal gyrus connectivity representing a potential pathogenic mechanism [45]. Given the observed memory decline in preeclampsia patients, particular attention should be directed toward cortical atrophy in the R-PoCg and R-SF – especially the R-PoCg – since such structural changes may compromise the function of neural conduction networks and consequently contribute to memory impairment.

Longitudinal studies have demonstrated that cortical thickness alterations associated with preeclampsia show significant recovery at follow-up compared to baseline measurements. However, no substantial changes in overall cognitive function levels have been observed. These findings suggest that the cortical thickness changes in preeclampsia may represent a transient compensatory mechanism, while cognitive impairments may persist over time and exhibit limited spontaneous recovery.

Findings from previous animal and clinical studies indicate that hippocampal GMV decreases during pregnancy and exhibits partial recovery approximately two years postpartum, potentially facilitating the restoration of pregnancy-related memory deficits [[46], [47], [48], [49]]. In this study, a longitudinal follow-up of the preeclampsia group three years later revealed a significant improvement in AVLT scores compared to baseline measurements, consistent with prior observations. Based on these findings, it is hypothesized that targeting hippocampal plasticity may offer novel insights into the understanding and intervention of memory impairment in patients with preeclampsia.

It is worth noting that future research may focus on the underlying mechanisms and clinical translation of cortical thickness alterations in preeclampsia. First, by leveraging the multimodal framework proposed by Lv, retinal imaging could enable non-invasive monitoring of brain atrophy dynamics [50]. Second, given the established association between retinal vasculature and cerebral small vessel disease, specific biomarkers may be identified to enhance early screening accuracy [51]. Furthermore, extending investigations to cerebrospinal fluid dynamics—such as the interaction between choroid plexus function and retinal parameters—could yield novel insights into neurovascular coupling. These research avenues collectively hold promise for advancing interdisciplinary innovation across multiple dimensions and improving comprehensive brain health monitoring.

Furthermore, resting-state fMRI studies conducted across human brain development have demonstrated that preeclampsia alters fetal resting-state functional connectivity (rs-FC). In children, rs-FC is increased between the right amygdala and the left frontal pole, between the left amygdala and bilateral frontal poles, and between the bilateral medial prefrontal cortex and the precuneus. Additionally, rs-FC is decreased between the bilateral medial prefrontal cortex and the left occipital fusiform gyrus [52]. Offspring of mothers with gestational hypertension exhibit structural and functional alterations in the frontal, parietal, and temporal regions during adolescence and are at increased risk for neurodevelopmental and psychiatric disorders, including depression, anxiety, autism spectrum disorder, and attention-deficit/hyperactivity disorder [53]. However, it remains controversial whether cortical thickness mediates the effect of maternal hypertensive disorders on offspring cognitive function [54]. This study will continue longitudinal follow-up of the preeclampsia, PHC, NPHC, and offspring groups to further investigate the pathophysiological mechanisms underlying preeclampsia-related cerebral cortical changes and associated cognitive decline.

This study has several limitations. First, the relatively small sample size limits the feasibility of more advanced machine learning analyses. Second, the study focused exclusively on brain gray matter structure and did not examine cerebral blood flow, cerebral oxygen metabolism, or functional connectivity. Third, genome-wide association study results identified 220 genetic loci linked to cortical thickness, with 95 demonstrating interactions between cognitive function and cardiovascular diseases [55]. These findings underscore the need for continued advancement and broader implementation of genetic testing in future research.

To the best of our knowledge, this study is the first to identify specific alterations in the cerebral cortex of patients with preeclampsia, including the cortical thickness of R-CACg, R-PoCg, R-RoACg, and R-SF, which were significantly reduced in the preeclampsia group compared to the PHC group. These structural changes were strongly associated with clinical factors such as elevated MAP, high pre-pregnancy BMI, and decreased scores on the MoCA and AVLT. These findings suggest that these cortical alterations may be involved in the pathophysiological mechanisms of cognitive impairment mediated by MAP and pre-pregnancy BMI. Furthermore, they may represent the structural basis underlying cognitive dysfunction in preeclampsia, providing insights into the microstructural evolution of the brain and its relationship with cognitive changes. It may represent one of the key early alterations in brain microstructure associated with Alzheimer's disease, highlighting the importance of carefully evaluating a patient's history of preeclampsia in clinical assessment and risk stratification. Longitudinal analyses have confirmed that preeclampsia is associated with reversible changes in cortical thickness and recoverable memory decline. However, no significant recovery in overall cognitive function or its associated serological markers has been observed to date.

## Data availability statement

The datasets used and/or analyzed during the current study are available from the corresponding author on reasonable request.

## Ethical approval

This prospective study was reviewed and approved by the Ethics Committee of Jinan Maternity and Child Health Hospital, Affiliated with Shandong First Medical University (Approval No. 20,190,618). All participants were recruited from the obstetric outpatient department, inpatient ward, and health examination center of the hospital between June 2021 and October 2023. These participants were provided with detailed information regarding the experimental procedures and safety precautions prior to enrollment and voluntarily signed the written informed consent form.

## Funding

This work was supported by grants from the Technology Development Plan of Jinan (202134072, 202225035), Science and Technology Project of Jinan Municipal Health Commission (2021289, 2021293) and Special Fund for Scientific and Technological Innovation of Shandong Maternal and Child Health Care Commission (Lu Fu You Xie Fa 202119).

## CRediT authorship contribution statement

**Yuanyuan Wang:** Writing – original draft, Software, Resources, Methodology, Investigation, Formal analysis, Data curation, Conceptualization. **Meng Li:** Formal analysis. **Tao Chen:** Data curation. **Yanli Li:** Data curation. **Qingqing Wang:** Data curation. **Xinyue Zhang:** Data curation. **Na Wang:** Data curation. **Linfeng Yang:** Funding acquisition. **Lingfei Guo:** Writing – review & editing. **Wenying Nie:** Data curation.

## Declaration of competing interest

The authors declare that they have no known competing financial interests or personal relationships that could have appeared to influence the work reported in this paper.

## Appendix Supplementary materials

Image, application 1

Image, application 2

Image, application 3

Image, application 4

Image, application 5
