# Supplementary material for: Preeclampsia as a reversible risk factor for Alzheimer’s disease: A prospective MRI study on morphological changes of the cerebral cortex and impairment of cognitive functions
Source: J Prev Alzheimers Dis. 2026 Jan 9;13(3):100475. doi: 10.1016/j.tjpad.2025.100475 (PMC12988370; doi:10.1016/j.tjpad.2025.100475)
Supplement: Supplementary file 1 [file mmc1.docx]

**Supplementary Material 1**

**Regarding the inclusion of both NPHC (Non-Pregnant Healthy Control) and PHC (Pregnant Healthy Control) groups as control conditions:**

We established the NPHC and PHC groups concurrently to more precisely isolate and identify changes specifically associated with pregnancy status and the pathophysiology of preeclampsia.

1. Role of the NPHC group (non-pregnant healthy control): This group serves as a baseline reference for assessing pregnancy-related effects. By comparing the PE group with the NPHC group, we were able to capture the combined effects of both "pregnancy" and "disease."
2. Role of the PHC group (pregnant healthy control group): This group is essential for accounting for the extensive physiological changes—including cognitive function and serum biomarker levels—associated with the "pregnancy" state itself. By comparing the PE group with the PHC group, we are able to isolate specific alterations that extend beyond the normal physiological range of pregnancy and are attributable to the pathophysiology of preeclampsia.

In summary, this study design allows us to demonstrate that the observed phenomenon is not only present in women with preeclampsia compared to non-pregnant women, but also significantly distinct from that in healthy pregnant women, thereby strengthening its specific association with preeclampsia rather than being attributable to normal pregnancy physiology alone.
