# Supplementary material for: Preeclampsia as a reversible risk factor for Alzheimer’s disease: A prospective MRI study on morphological changes of the cerebral cortex and impairment of cognitive functions
Source: J Prev Alzheimers Dis. 2026 Jan 9;13(3):100475. doi: 10.1016/j.tjpad.2025.100475 (PMC12988370; doi:10.1016/j.tjpad.2025.100475)
Supplement: Supplementary file 2 [file mmc2.docx]

**Supplementary Material 2**

Participant attrition in longitudinal studies is indeed a common challenge that may introduce potential bias. In our study, the reasons for participant loss between the initial cohort of 111 individuals and the final sample of 20 completed follow-ups were multifaceted. The most prevalent reason was loss of contact, accounting for approximately 54.05% of all dropouts. The primary contributing factors include:

1. High mobility of study participants: A substantial number of participants were of reproductive age and experienced significant life transitions during the follow-up period—such as relocation after childbirth, job changes, or updated phone numbers—making it difficult to re-establish communication.

2. Inaccurate or outdated contact information: Despite our best efforts to maintain current contact details, some information became obsolete over the three-year follow-up period.

3. Refusal to continue participation: This accounted for approximately 44% of the attrition. Commonly reported reasons included:

(1) Time constraints or perceived burden: Some participants indicated that the follow-up assessments were time-consuming or burdensome, particularly during the postpartum period when caregiving responsibilities are high.

(2) Loss of interest or diminished motivation: A subset of participants stated that their primary motivation for enrollment was related to the perinatal phase, and they subsequently lost interest in long-term follow-up.

We have expanded the description of these issues in the "Methods" section of the revised manuscript, and the exclusion process along with detailed information on the 36 participants lost during follow-up is now presented in Figure 1. To assess the potential impact of attrition, we conducted a sensitivity analysis comparing baseline characteristics—including maternal age, blood pressure, and key biochemical indicators—between the 20 participants who completed follow-up and those who did not. The results revealed no statistically significant differences in these core baseline variables (see Supplementary Material new Table1), suggesting that the missing data are likely missing at random with respect to these factors. This strengthens our confidence that participant dropout is unlikely to have introduced substantial bias into the observed associations related to these baseline characteristics.

**Table new S1.** The basic characteristics of the participants in the follow-up study

| Characteristic | Base line  (n=20) | Follow-up  (n=20) | *t*/Z | *P* |
| --- | --- | --- | --- | --- |
| Age (Year) | 31.14±5.20 | 30.35±4.18 | 0.645^a^ | 0.520 |
| Gestational age（weeks） | 32.76±4.83 | 32.39±5.24 | 0.313^a^ | 0.755 |
| Systolic pressure (mmHg) | 157.13±14.95 | 157.85±12.24 | -0.522^b^ | 0.602 |
| Diastolic pressure (mmHg) | 99.62±10.63 | 103.45±13.30 | -0.890^b^ | 0.373 |
| Mean atrial pressure (mmHg) | 118.79±10.77 | 121.60±12.33 | -0.614^b^ | 0.539 |
| Hemoglobin (g/L) | 125.70±11.52 | 127.15±13.21 | -0.439^b^ | 0.661 |
| Platelet count (×10^9^/L) | 219.55±64.70 | 182.80±45.94 | 2.429^a^ | 0.017 |
| Creatinine (μmol/L) | 53.14±14.77 | 56.00±13.51 | -0.890^b^ | 0.373 |

**Note:**

Data are mean ± standard deviation;

^a^：Pared-samples T test, *t*；^b^：Paired samples rank sum test, *Z*
