# Supplementary material for: Preeclampsia as a reversible risk factor for Alzheimer’s disease: A prospective MRI study on morphological changes of the cerebral cortex and impairment of cognitive functions
Source: J Prev Alzheimers Dis. 2026 Jan 9;13(3):100475. doi: 10.1016/j.tjpad.2025.100475 (PMC12988370; doi:10.1016/j.tjpad.2025.100475)
Supplement: Supplementary file 3 [file mmc3.docx]

**Supplementary Materials 3**

**Detailed description of the standardized implementation procedure for neuropsychological assessment**

The following outlines the specific implementation procedures of the neuropsychological assessment, designed to ensure consistency and effective quality control throughout the assessment process：

1. **Assessment of environmental standardization and control**

To ensure the comparability and reliability of the assessment results, we implemented strict standardization of the testing environment.

**(1) Physical Environment Requirements:** The assessment must be conducted in a dedicated neuropsychological testing room. This environment should ensure quietness, adequate ventilation, and appropriate lighting to prevent auditory distractions, thermal discomfort, or suboptimal visual conditions from affecting participants' attention and emotional state. The testing room should be situated away from high-traffic or noisy areas such as phlebotomy rooms and waiting halls to minimize external disturbances as much as possible.

**(2) Space and Personnel Allocation:** The assessment is typically conducted in a setting with only the examiner and the participant present, to prevent unintended cues or distractions from family members or bystanders. For participants requiring special assistance, a supportive companion may be permitted to remain in the room; however, the companion must be explicitly instructed not to participate in or provide any form of assistance during the assessment process.

**(3) Equipment and Materials:** All assessments were conducted using standardized test materials. Each set was cleaned before and after use, and routine inspections were performed to monitor wear and tear, ensuring consistency in material specifications.

**2. Examiner Qualifications and Systematic Training**

The professional competence of examiners is critical to ensuring assessment quality, and substantial emphasis has been placed on this aspect.

1. **Professional Qualification Requirements:** All examiners involved in the assessment must hold a background in neuropsychology, psychology, or related disciplines and have completed systematic training in neuropsychological assessment. The assessment team comprises neurologists with expertise in brain-cognition-behavior relationships and demonstrated proficiency in standardized testing methodologies.
2. **Standardized Training and Calibration:** Prior to data collection, all examiners underwent centralized training on the neuropsychological scales employed in this study. The training utilized a "situational teaching approach," simulating authentic clinical testing scenarios, and was delivered by experienced neuropsychological experts who provided detailed explanations and live demonstrations. Following this, examiners practiced in pairs under on-site supervision by the experts. A critical component of the process was the "consistency check": pre-recorded standardized assessment videos were presented to all examiners, who independently scored each item and compared their results with the expert-provided reference scores. For items exhibiting score discrepancies—particularly complex items such as "abstract reasoning" and "cube copying"—in-depth discussions and supplementary training sessions were conducted to ensure examiner scores achieved high agreement with the reference standards (Kappa value > 0.85).
3. **Continuous Quality Control and Supervision:** Throughout the research process, consistency calibration is conducted periodically (e.g., every two months) to prevent temporal drift in assessment standards. Concurrently, a senior researcher conducts random audits of audio and video recordings of the assessment sessions, as well as scoring records, to ensure ongoing consistency in procedural implementation and scoring criteria across the entire data collection period.
4. **Standardized Assessment Procedures and Participant Preparation**

Standardized procedures are essential for minimizing random errors：

**(1) Establishing a Rapport:** Prior to the assessment, the examiner engages in a brief, friendly, and empathetic conversation with the participant to explain the purpose and importance of the evaluation, alleviate anxiety, uncertainty, or apprehension, and establish a cooperative and trusting relationship.

**(2) Assessing the Participant's Condition:** Prior to the formal assessment, the examiner verifies that the participant is free from physical discomfort, such as headache, dizziness, or fever. Should the participant experience acute symptoms or an epileptic seizure during testing, the assessment will be immediately suspended and the incident documented. Testing will be rescheduled only after the participant has fully recovered.

**(3) Standardized Instructions:** All assessments are conducted in strict accordance with the operational manuals of each scale, using standardized and consistent instructions. No additional or extraneous content is introduced beyond the prescribed protocols. For timed tests, a stopwatch is employed to ensure precise time measurement.

**(4) Assessment Sequence and Pacing:** The evaluation of cognitive functions follows a standardized sequence. Tasks assessing attention and language are typically administered first, while memory function tests are scheduled toward the end to minimize interference from proactive inhibition. Each testing session lasts no more than 30 minutes to prevent participant fatigue. For assessments with extended total duration, multiple breaks (approximately 10 minutes each) are incorporated, and the assessment is completed across separate time intervals.

**4. Quality Control Management for Data Recording and Result Interpretation**

A standardized data processing procedure serves as the final step in ensuring the scientific rigor of the results.

**(1) Data Recording and Verification:** The original scores from all evaluations were promptly and accurately recorded on standardized case report forms. Prior to database entry, all data underwent 100% verification by an independent researcher who was not involved in the assessment process, to ensure consistency between the original records and the entered data.

**(2) Result Analysis and Reporting:** The interpretation of neuropsychological assessment results is not conducted in isolation but requires collaborative input from a multidisciplinary team, including neurologists and medical psychology specialists. Interpretation is based on an integrative discussion that considers the patient’s medical history, clinical manifestations, and neuroimaging findings, leading to the formulation of final conclusions and recommendations. The assessment report includes not only standardized test scores but also a description of the participant’s level of cooperation, emotional state, motivation, and other behavioral observations that may influence test performance.
