# Supplementary material for: Preeclampsia as a reversible risk factor for Alzheimer’s disease: A prospective MRI study on morphological changes of the cerebral cortex and impairment of cognitive functions
Source: J Prev Alzheimers Dis. 2026 Jan 9;13(3):100475. doi: 10.1016/j.tjpad.2025.100475 (PMC12988370; doi:10.1016/j.tjpad.2025.100475)
Supplement: Supplementary file 4 [file mmc4.docx]

**Supplementary Materials 4**

**Image Acquisition:**

1 Anatomical structure images were obtained using a 3D-T_1_WI (T_1_-weighted image, T_1_WI) sequence for high-resolution three-dimensional brain MRI data: repetition time (TR) = 25 ms, echo time (TE) = 6.1 ms, inversion time (TI) = 900 ms, flip angle = 30°, voxel size = 1 mm³.

2 Three-dimensional multi-echo gradient-echo (3D ME-GRE) sequence: TR = 50 ms, initial TE = 5 ms, TE interval = 9 ms, number of echoes = 5, flip angle = 20°, voxel size = 1 × 1 × 0.9 mm³.

3 T_2_-weighted imaging (T_2_WI) (TR = 3700 ms, TE = 109 ms, flip angle = 15°, slice thickness = 5 mm), T_2_WI-FLAIR (TR = 8000 ms, TE = 81 ms, flip angle = 15°, slice thickness = 5 mm), and diffusion-weighted imaging (TR = 3900 ms, TE = 65 ms, flip angle = 180°, slice thickness = 5 mm), which were applied to screen for potential cerebral abnormalities.
