# Supplementary material for: Preeclampsia as a reversible risk factor for Alzheimer’s disease: A prospective MRI study on morphological changes of the cerebral cortex and impairment of cognitive functions
Source: J Prev Alzheimers Dis. 2026 Jan 9;13(3):100475. doi: 10.1016/j.tjpad.2025.100475 (PMC12988370; doi:10.1016/j.tjpad.2025.100475)
Supplement: Supplementary file 5 [file mmc5.docx]

**Table S1**. The clinical characteristics

| **Characteristic** | **NPHC**  (n=77) | **PHC**  (n=26) | **Preeclampsia**  (n=111) | *F /t /χ2* | *P* | **Post hoc test** | | |
| --- | --- | --- | --- | --- | --- | --- | --- | --- |
|  |  |  |  |  |  | a | b | c |
| **Signs** **on day of first assessment** | | | | | | | | |
| Age (Year) | 32.21±5.66 | 31.04±5.21 | 31.14±5.20 | 1.006 ^a^ | 0.367 | - | - | - |
| Height (cm) | 162.03±4.30 | 162.96±4.65 | 161.15±5.43 | 1.681 ^a^ | 0.189 | - | - | - |
| Weight (Kg) | 59.09±8.33 | 71.55±8.71 | 81.89±12.12 | - | - | - | - | - |
| Body Mass Index (BMI) (Kg/m^2^) | 22.53±3.17 | - | - | - | - | - | - | - |
| Systolic pressure (mmHg) | 111.49±9.80 | 113.69±12.16 | 157.13±14.95 | 322.056 ^a^ | ＜0.001 | 0.456 | ＜0.001 | ＜0.001 |
| Diastolic pressure (mmHg) | 69.01±8.58 | 74.35±9.96 | 99.62±10.63 | 238.242 ^a^ | ＜0.001 | 0.018 | ＜0.001 | ＜0.001 |
| Mean atrial pressure (mmHg) | 83.17±8.32 | 87.46±9.93 | 118.79±10.77 | 330.040 ^a^ | ＜0.001 | 0.056 | ＜0.001 | ＜0.001 |
| **Past and current medical and obstetrical history** | | | | | | | | |
| Gestational age（weeks） | - | 32.82±5.41 | 32.76±4.83 | 0.052 ^b^ | 0.959 | - | - | - |
| Reproductive history（GPL） | - | G 2.45±1.60  P 0.82±1.05  L 0.73±0.88 | G 2.38±1.41  P 0.50±0.63  L 0.48±0.60 | 0.226 ^b^  1.350 ^b^  1.270 ^b^ | 0.822  0.189  0.216- | - | - | - |
| Pre-pregnancy weigh (Kg) | - | 59.65±9.85 | 68.98±12.82 | -3.473 ^b^ | 0.001 | - | - | - |
| Pre-pregnancy BMI (Kg/m^2^) | - | 22.45±3.44 | 26.54±4.58 | -4.267 ^b^ | ＜0.001 | - | - | - |
| Symptoms on day of first assessment | | | | | | | | |
| Anemia n (%) | - | 4 (15.4%) | 13 (17.7%) | -0.261 ^c^ | 0.609 | - | - | - |
| Dizziness n (%) | - | 2 (7.7%) | 6 (5.4%) | 0.200 ^c^ | 0.654 | - | - | - |
| Headache n (%) | - | 1 (3.9%) | 20 (18.0%) | 3.260 ^c^ | 0.071 | - | - | - |
| Nodal tachycardia n (%) | - | 1 (3.9%) | 6 (5.4%) | 0.106 ^c^ | 0.745 | - | - | - |
| Placental abruption n (%) | - | 1 (3.9%) | 1 (0.9%) | 1.270 ^c^ | 0.260 | - | - | - |
| Umbilical cord around the neck n (%) | - | 6 (23.1%) | 38 (34.2%) | 1.203 ^c^ | 0.273 | - | - | - |
| Fetal growth restriction n (%) | - | 0 | 22 (19.8%) | 6.139 ^c^ | 0.013 | - | - | - |
| **Laboratory tests—worst values on day of first assessment** | | | | | | | | |
| Serum T-tau (pg/ml) | 1344.88±561.83 | 1347.50±650.09 | 1379.06±631.27 | 0.081 | 0.922 | - | - | - |
| Serum P-tau181 (pg/ml) | 73.43±36.83 | 71.48±48.40 | 89.58±66.92 | 2.399 | 0.093 | - | - | - |
| Serum Aβ1-42 (pg/ml) | 54.29±28.98 | 51.68±23.97 | 80.37±48.23 | 12.112 | ＜0.001 | 0.733 | ＜0.001 | 0.001 |
| Hemoglobin (g/L) | 126.84±10.89 | 116.46±11.14 | 125.70±11.52 | 8.736 | ＜0.001 | ＜0.001 | 0.495 | ＜0.001 |
| Platelet count (×10^9^/L) | 236.36±61.78 | 217.12±51.14 | 219.55±64.70 | 1.919 | 0.149 | - | - | - |
| Creatinine (μmol/L) | 48.17±11.70 | 41.08±8.67 | 53.14±14.77 | 9.932 | ＜0.001 | 0.018 | 0.011 | ＜0.001 |
| **Neuropsychological assessment (score)** | | | | | | | | |
| Education (year) | 16.57±1.63 | 14.73±2.05 | 13.46±2.40 | 49.438 | ＜0.001 | ＜0.001 | ＜0.001 | 0.006 |
| Montreal Cognitive Assessment (MoCA) | 29.75±0.65 | 29.12±1.34 | 28.05±1.62 | 38.648 | ＜0.001 | 0.034 | ＜0.001 | ＜0.001 |
| Symbol Digit Modalities Test (SDMT) | 59.78±7.75 | 52.92±8.39 | 49.24±9.07 | 34.705 | ＜0.001 | ＜0.001 | ＜0.001 | 0.049 |
| Auditory Word Learning Test (AVLT) | 46.42±10.58 | 41.62±10.39 | 35.00±7.65 | 35.925 | ＜0.001 | 0.022 | ＜0.001 | 0.001 |
| Stroop Color Word Test (SCWT) | 82.06±13.64 | 93.65±16.19 | 99.32±18.79 | 24.074 | ＜0.001 | 0.003 | ＜0.001 | 0.123 |
| **Symptoms of the newborn at birth** | | | | | | | | |
| Apgar score（1min) | - | 9.29±1.69 | 8.98±1.43 | 0.816 ^b^ | 0.416 | - | - | - |

**Note:**

Data are mean ± standard deviation;

Post hoc test: a = NPHC vs PHC; b = NPHC vs Preeclampsia; c = PHC vs Preeclampsia;

^a^: One-way analysis of variance；^b^：Two independent samples t-test；^c^：Chi-squared test

Auditory Verbal Learning Test: Auditory Verbal Learning Test-Huashan version

Stroop Color Word Test: Sum of timing for cards 2 and 3

**Table S2**. The thickness differences of 68 sub-regions of the cerebral cortex among the three groups (positive results section)

| **Characteristic** | **NPHC**  (n=78) | **PHC**  (n=26) | **Preeclampsia** (n=111) | *F* | *P** | **Post hoc test** | | |
| --- | --- | --- | --- | --- | --- | --- | --- | --- |
|  |  |  |  |  |  | a | b | c |
| **Cortical thickness of first measurement** | | | | | | | | |
| L- Entorhinal (En) | 1.37±0.15 | 1.50±0.18 | 1.47±0.18 | 10.038 | ＜0.001 | 0.001 | ＜0.001 | 0.346 |
| L- Lingual (Lg) | 1.40±0.08 | 1.46±0.10 | 1.44±0.09 | 9.347 | ＜0.001 | 0.001 | ＜0.001 | 0.287 |
| L- Pars Opercularis (Op) | 1.98±0.11 | 1.93±0.11 | 1.93±0.11 | 5.599 | 0.004 | 0.034 | 0.002 | 0.969 |
| L- Pericalcarine (PerCa) | 1.16±0.13 | 1.23±0.08 | 1.23±0.14 | 6.742 | 0.001 | 0.029 | ＜0.001 | 0.892 |
| L- Temporal Pole (Tpol) | 1.58±0.41 | 1.75±0.32 | 1.75±0.33 | 5.323 | 0.006 | 0.042 | 0.002 | 0.999 |
| R- Caudal Anterior Cingulate (CACg) | 1.70±0.11 | 1.76±0.09 | 1.71±0.11 | 3.556 | 0.030 | 0.016 | 0.923 | 0.010 |
| R-En | 1.36±0.16 | 1.47±0.16 | 1.46±0.15 | 10.177 | ＜0.001 | 0.002 | ＜0.001 | 0.728 |
| R-Lg | 1.38±0.08 | 1.42±0.07 | 1.41±0.09 | 3.733 | 0.026 | 0.030 | 0.020 | 0.498 |
| R-PerCa | 1.10±0.10 | 1.17±0.10 | 1.16±0.13 | 7.625 | 0.001 | 0.005 | ＜0.001 | 0.593 |
| R- Posterior Cingulate (PoCg) | 1.68±0.08 | 1.73±0.08 | 1.67±0.08 | 5.678 | 0.004 | 0.014 | 0.247 | 0.001 |
| R- Rostral Anterior Cingulate (RoACg) | 1.76±0.15 | 1.87±0.15 | 1.77±0.12 | 7.627 | 0.001 | ＜0.001 | 0.369 | 0.001 |
| R- Superior Frontal (SF) | 1.96±0.12 | 1.97±0.11 | 1.92±0.12 | 4.787 | 0.009 | 0.711 | 0.008 | 0.028 |
| R-Tpol | 1.51±0.34 | 1.69±0.30 | 1.68±0.30 | 7.255 | 0.001 | 0.010 | ＜0.001 | 0.786 |
| R- Insula (Ins) | 1.73±0.12 | 1.78±0.11 | 1.77±0.10 | 3.435 | 0.034 | 0.041 | 0.023 | 0.562 |
| **Cortical surface area of first measurement** | | | | | | | | |
| L- Medial Orbitofrontal (MOrF) | 2730.51±440.74 | 2556.69±294.98 | 2592.65±348.60 | 3.687 | 0.027 | 0.044 | 0.015 | 0.664 |
| L- Para Central (PaC) | 2533.62±419.74 | 2251.58±267.81 | 2336.00±342.62 | 9.013 | ＜0.001 | 0.001 | ＜0.001 | 0.290 |
| R- Transverse Temporal (TrT) | 554.18±110.93 | 508.46±104.57 | 517.35±89.72 | 3.769 | 0.025 | 0.044 | 0.013 | 0.683 |

**Note:**

*: Tukey’s honestly significant difference post hoc test was used for pairwise comparisons between groups, whereas the least significant difference method was applied for multiple comparisons correction;

Data are mean ± standard deviation;

Post hoc test: a = NPHC vs PHC; b = NPHC vs Preeclampsia; c = PHC vs Preeclampsia;

L, left; R, right.

**Table S3**. Person correlation analysis of the thickness and surface area of the cerebral cortex and clinical characteristics

| **Brain regions VS**  **clinical characteristics** | **MAP** | | |  | **Pre-pregnancy BMI** | |  | **Serum Aβ1-42** | |  | **Serum P-tau181** | |
| --- | --- | --- | --- | --- | --- | --- | --- | --- | --- | --- | --- | --- |
|  | *r* | *P* | |  | *r* | *P* |  | *r* | *P* |  | *r* | *P* |
| **Cortical thickness of first** **assessment** | | | | | | | | | | | | |
| L-En | 0.252 | | ＜0.001 |  | -0.021 | 0.757 |  | -0.013 | 0.845 |  | -0.054 | 0.428 |
| L-Lg | 0.140 | | 0.041 |  | 0.156 | 0.022 |  | 0.097 | 0.158 |  | 0.060 | 0.379 |
| L-Op | -0.190 | | 0.005 |  | -0.224 | 0.001 |  | -0.061 | 0.376 |  | -0.087 | 0.203 |
| L-PerCa | 0.165 | | 0.016 |  | 0.208 | 0.002 |  | -0.008 | 0.912 |  | -0.011 | 0.875 |
| L-Tpol | 0.164 | 0.016 | |  | -0.022 | 0.748 |  | -0.116 | 0.090 |  | -0.092 | 0.181 |
| R-CACg | -0.029 | 0.672 | |  | -0.190 | 0.005 |  | -0.095 | 0.166 |  | -0.110 | 0.108 |
| R-En | 0.214 | 0.002 | |  | -0.041 | 0.550 |  | -0.047 | 0.497 |  | -0.082 | 0.234 |
| R-Lg | 0.073 | 0.287 | |  | 0.119 | 0.083 |  | 0.099 | 0.150 |  | 0.110 | 0.109 |
| R-PerCa | 0.165 | 0.015 | |  | 0.217 | 0.001 |  | 0.005 | 0.946 |  | -0.068 | 0.319 |
| R-PoCg | -0.169 | 0.013 | |  | -0.131 | 0.055 |  | -0.043 | 0.529 |  | -0.021 | 0.762 |
| R-RoACg | -0.053 | 0.442 | |  | -0.186 | 0.006 |  | -0.037 | 0.587 |  | -0.035 | 0.609 |
| R-SF | -0.207 | 0.002 | |  | -0.253 | ＜0.001 |  | -0.120 | 0.080 |  | -0.120 | 0.079 |
| R-Tpol | 0.117 | 0.087 | |  | -0.082 | 0.235 |  | -0.017 | 0.805 |  | -0.040 | 0.565 |
| R-Ins | 0.059 | 0.392 | |  | -0.052 | 0.449 |  | -0.056 | 0.418 |  | -0.014 | 0.842 |
| **Cortical surface area of first assessment** | | | | | | | | | | | | |
| L-MOrF | -0.080 | 0.246 | |  | 0.033 | 0.626 |  | -0.046 | 0.507 |  | -0.035 | 0.608 |
| L-PaC | -0.138 | 0.044 | |  | 0.015 | 0.823 |  | -0.054 | 0.429 |  | -0.040 | 0.562 |
| R-TrT | -0.080 | 0.241 | |  | -0.017 | 0.805 |  | -0.052 | 0.449 |  | -0.015 | 0.829 |
| **Brain regions VS Neuropsychological tests** | **MoCA** | | |  | **SDMT** | |  | **AVLT** | |  | **SCWT** | |
|  | *r* | *P* | |  | *r* | *P* |  | *r* | *P* |  | *r* | *P* |
| **Cortical thickness of first assessment** | | | | | | | | | | | | |
| L-En | -0.045 | 0.516 | |  | -0.090 | 0.191 |  | -0.055 | 0.421 |  | 0.125 | 0.068 |
| L-Lg | -0.171 | 0.012 | |  | -0.137 | 0.045 |  | -0.063 | 0.362 |  | 0.052 | 0.451 |
| L-Op | 0.174 | 0.011 | |  | 0.250 | 0.000 |  | 0.183 | 0.007 |  | -0.132 | 0.053 |
| L-PerCa | -0.206 | 0.002 | |  | -0.191 | 0.005 |  | -0.170 | 0.013 |  | 0.118 | 0.085 |
| L-Tpol | -0.080 | 0.245 | |  | -0.068 | 0.323 |  | -0.140 | 0.040 |  | 0.104 | 0.128 |
| R-CACg | -0.023 | 0.736 | |  | -0.029 | 0.672 |  | -0.074 | 0.284 |  | -0.040 | 0.563 |
| R-En | -0.114 | 0.096 | |  | -0.056 | 0.416 |  | -0.069 | 0.316 |  | 0.161 | 0.019 |
| R-Lg | -0.064 | 0.351 | |  | -0.018 | 0.794 |  | -0.015 | 0.826 |  | -0.014 | 0.840 |
| R-PerCa | -0.130 | 0.057 | |  | -0.130 | 0.057 |  | -0.120 | 0.079 |  | 0.134 | 0.050 |
| R-PoCg | 0.076 | 0.266 | |  | 0.029 | 0.668 |  | 0.189 | 0.006 |  | -0.093 | 0.176 |
| R-RoACg | -0.010 | 0.884 | |  | -0.074 | 0.279 |  | -0.057 | 0.409 |  | 0.056 | 0.412 |
| R-SF | 0.137 | 0.046 | |  | 0.120 | 0.081 |  | 0.148 | 0.030 |  | -0.062 | 0.366 |
| R-Tpol | -0.084 | 0.219 | |  | -0.007 | 0.916 |  | -0.046 | 0.499 |  | 0.135 | 0.048 |
| R-Ins | -0.033 | 0.634 | |  | -0.010 | 0.882 |  | 0.078 | 0.257 |  | 0.085 | 0.215 |
| **Cortical surface area of first assessment** | | | | | | | | | | | | |
| L-MOrF | -0.054 | 0.434 | |  | 0.065 | 0.347 |  | 0.034 | 0.619 |  | -0.056 | 0.412 |
| L-PaC | -0.004 | 0.958 | |  | 0.050 | 0.466 |  | 0.107 | 0.120 |  | -0.127 | 0.064 |
| R-TrT | 0.064 | 0.351 | |  | 0.098 | 0.155 |  | 0.065 | 0.346 |  | -0.104 | 0.130 |

**Note:**

MAP: Mean atrial pressure (mmHg);

MoCA: Montreal Cognitive Assessment (score);

Pre-pregnancy BMI: Pre-pregnancy Body Mass Index (Kg/m2);

Serum Aβ1-42: Serum Aβ1-42 concentration (pg/ml);

Serum P-tau181: Serum P-tau181 concentration (pg/ml);

MoCA: Montreal Cognitive Assessment;

SDMT: Symbol Digit Modalities Test;

AVLT: Auditory Word Learning Test;

SCWT: Stroop Color Word Test.

**Table S4.** The longitudinal changes in the thickness of the cerebral cortex, laboratory tests, and neuropsychological assessments in the preeclampsia group

| **Characteristic** | **Base line**  (n=20) | **Follow-up**  (n=20) | *t*/Z/*r* | *P* |
| --- | --- | --- | --- | --- |
| **Cortical thickness** | | | | |
| R-CACg | 1.706 ± 0.105 | 1.794 ± 0.250 | -3.382 ^b^ | 0.001 |
| R-PoCg | 1.640 ± 0.079 | 1.794 ± 0.119 | -4.836 ^a^ | ＜0.001 |
| R-RoACg | 1.754 ± 0.135 | 1.928 ± 0.240 | -2.831 ^a^ | 0.007 |
| R-SF | 1.876 ± 0.128 | 1.982 ± 0.232 | -3.571 ^b^ | ＜0.001 |
| **Laboratory and Neuropsychological tests** | | | | |
| Serum Aβ1-42 (pg/ml) | 71.110±43.350 | 84.100±27.450 | -1.929 ^b^ | 0.054 |
| MoCA (score) | 29.000±2.000 | 28.000±2.000 | -1.046 ^b^ | 0.295 |
| SDMT (score) | 49.800±9.275 | 55.933±7.592 | -1.982 ^a^ | 0.057 |
| AVLT (score) | 33.800±5.882 | 63.133±11.064 | -9.067 ^a^ | ＜0.001 |
| **Correlation analysis** | | | | |
| R-CACg - Serum Aβ1-42 (pg/ml) | - | - | -0.246 ^c^ | 0.376 |
| R-CACg - MoCA | - | - | 0.424 ^d^ | 0.116 |
| R-CACg - SDMT | - | - | 0.336 ^d^ | 0.220 |
| R-CACg - AVLT | - | - | 0.067 ^d^ | 0.813 |
| R-PoCg - Serum Aβ1-42 (pg/ml) | - | - | -0.175 ^c^ | 0.533 |
| R-PoCg - MoCA | - | - | 0.408 ^d^ | 0.131 |
| R-PoCg - SDMT | - | - | 0.259 ^d^ | 0.352 |
| R-PoCg - AVLT | - | - | 0.083 ^d^ | 0.768 |
| R-RoACg - Serum Aβ1-42 (pg/ml) | - | - | -0.595 ^c^ | 0.019 |
| R-RoACg -MoCA | - | - | 0.416 ^d^ | 0.123 |
| R-RoACg - SDMT | - | - | 0.307 ^d^ | 0.266 |
| R-RoACg - AVLT | - | - | 0.096 ^d^ | 0.733 |
| R-SF - Serum Aβ1-42 (pg/ml) | - | - | -0.375 ^c^ | 0.168 |
| R-SF - MoCA | - | - | 0.255 ^d^ | 0.360 |
| R-SF - SDMT | - | - | 0.254 ^d^ | 0.362 |
| R-SF - AVLT | - | - | 0.273 ^d^ | 0.325 |

**Note:**

Serum Aβ1-42: Serum Aβ1-42 concentration (pg/ml);

MoCA: Montreal Cognitive Assessment (score);

MoCA: Montreal Cognitive Assessment;

SDMT: Symbol Digit Modalities Test;

AVLT: Auditory Word Learning Test;

^a^：Pared-samples T test；^b^：Paired samples rank sum test; ^c^: Spearman's bivariate correlation analysis; ^d^: Pearson's bivariate correlation analysis.
